# Quality of life in adult patients with congenital heart disease: Results of a double-center study

**DOI:** 10.3389/fpsyt.2022.1062386

**Published:** 2023-01-12

**Authors:** Zahra Khajali, Amin Sayyadi, Zahra Ansari, Maryam Aliramezany

**Affiliations:** ^1^Shaheed Rajaei Cardiovascular Medical and Research Center, Tehran, Iran; ^2^Student Research Committee, School of Medicine, Kerman University of Medical Sciences, Kerman, Iran; ^3^Cardiovascular Research Center, Afshar Hospital, Shahid Sadoughi University of Medical Sciences, Yazd, Iran; ^4^Cardiovascular Research Center, Institute of Basic and Clinical Physiology Sciences, Kerman University of Medical Sciences, Kerman, Iran

**Keywords:** congenital heart disease, quality of life, mental health, adult, QoL

## Abstract

**Introduction:**

Prevalence of congenital heart disease (CHD) has increased in recent years, and patients with CHD have to deal with sequelae of the multiple medical and surgical treatments that can affect different aspects of their life which could be reflected in their quality of life (QoL). In Iran, to the best of our knowledge, QoL of adult patients with CHD has not been investigated, so this study aimed to investigate the QoL of adult patients with CHD referred to two Iranian outpatient settings.

**Methods:**

In 2022, a double center, cross-sectional study was performed on adult patients with CHD receiving out-patient care at Besat Clinic in Kerman, Iran, and Clinic of Shahid Rajaee Hospital in Tehran, Iran. Inclusion criteria were documented diagnosis of CHD based on guidelines, ejection fraction of above 45% and age of 18–55 years. We recruited a sample of 120 individuals using a simple random sampling method. At the day of referring to the adult congenital heart diseases clinic, after ensuring their written consent to participate in the study, we asked patients to fill in two questionnaires of demographic questionnaire and Persian version of the Ferrans and Powers Quality of Life Index. Data were analyzed using descriptive statistics and chi square via SPSS-22.

**Results:**

A total of 101 patients with a mean age of 31.05 years participated in the study. Demographic and socio-economic factors had no significant relationship with the patients’ QoL. But factors related to the disease were significantly different among QoL groups (*P*-value > 0.05).

**Discussion:**

Adults with CHD had a low QoL, which is not related to their demographic and socio-economic factors. That is, the existence of the disease alone and its accompanying complications can affect the QoL of these people. Hence, the mental health of adult patients with CHD should be monitored during their routine cardiac care.

## Background

Congenital heart diseases (CHD) are on the rise globally. The total number of patients living with CHD has increased from 10,105,235 in 1990 to 11,998,283 in 2017, an increase of 187%. Moreover, the birth prevalence of CHD has increased by 4.2% (17,876 cases per 100,000 babies) (1). Recent diagnostic and therapeutic advances have also increased the number of adults with CHD (2–4). Furthermore, the prevalence of CHD differs among different regions and countries (5), but what is common among adults with CHD is the fact that they all have to deal with sequelae such as heart failure (HF), endocarditis, arrhythmias, and pulmonary hypertension, which can result from multiple medical and surgical treatments received in previous years (6, 7).

Furthermore, studies showed that psychiatric disorders such as anxiety and mood disorders can affect the quality of life in adult patients with congenital heart disease (8). These disorders can negatively affect patients’ employment and social relationships (9). Therefore, screening for psychiatric disorders such as major depressive disorders can help improve the patient’s condition (10).

Furthermore, these patients might also experience mental disorders. Lebherz et al. (6) reported an increased risk of anxiety disorders, regardless of the severity of the heart defect. To show the magnitude, they suggested that it is comparable to the anxiety level of patients with aggressive non-Hodgkin lymphoma. Gleason et al. noted elevated anxiety and depressive symptoms and believed that this combination is associated with unemployment and lower quality of life (QoL) (11).

The World Health Organization (WHO) defined QoL as “an individual’s perception of their position in life in the context of the culture and value systems in which they live and in relation to their goals, expectations, standards, and concerns” (12). For most people, QoL matters more than longevity (13). QoL is an important issue in the field of medicine, but measuring it scientifically is challenging because of its inherent subjectivity (14, 15). Scientists have introduced multiple assessment tools for measuring QoL. Spitzer et al. developed the Quality of Life Index (QLI), which assesses the physical, psychological, and social functioning of patients and yields a score from 0 to 10. The unique feature of the QLI is that it uses multiple resources, including the patient, the physician or other health professional, a relative, or others, and compares the results. Studies in Australia, Canada, and the United States have demonstrated its validity and reliability. Padilla’s Quality of Life Index (1983) is another tool focused on patients’ physical conditions, activities, and attitudes. It was originally developed for patients with cancer, but later, they also used it for patients with colostomy with some disease-specific added items (16).

Truong et al. (17) conducted a cross-sectional study on 109 hospitalized patients with CHD in Vietnam to investigate the QoL of adults. They used the EuroQOL-5D, the Satisfaction with Life Scale, and the Hospital Anxiety and Depression Scale as assessment tools and found that reduced QOL and elevated psychological problems were common among these patients. Unmarried status, unemployment/unstable employment, and complex CHD/pulmonary artery hypertension were found to be related to poor QOL, life dissatisfaction, anxiety, and depression. Rometsch et al. (18) investigated medical and psychosocial risk factors for impaired health-related quality of life (HRQoL) and poor psychological adjustment (PA) in young adults with CHD; their study included 188 patients with CHD and 139 healthy patients, and they discovered that these individuals had impaired physical HRQoL but normal mental HRQoL and PA; they also reported that lower physical exercise capacity, female sex, less social support, and a lower educational level are related to poor QoL.

Various studies proved the negative effects of various heart diseases on patients’ quality of life. For example, it has been shown that heart failure, regardless of the severity of the symptoms, can be a factor that influences the quality of life in an age-dependent manner (19). In addition, the quality of life of patients with coronary artery disease is an important indicator of their prognosis (20). Although the quality of life of patients with congenital heart disease has been investigated in previous studies (17, 18, 20–23), there is no consensus on it. QoL is a multifaceted concept in different countries; age, marital and employment status, and functional class assessment are some of the factors that can be responsible for this diversity (13, 14). To the best of our knowledge, QoL among patients with CHD has not been investigated in Iran. Therefore, considering the rise in the number of patients with CHD and the interaction of mental and physical health, this study aimed to investigate the QoL of adult patients with CHD referred to two Iranian outpatient clinics in 2022.

## Materials and methods

In 2022, a two-center study was designed on CHD patients receiving outpatient care from the Besat clinic in Kerman, Iran, and the clinic of the Shahid Rajaee hospital in Tehran, Iran. The inclusion criteria were documented diagnoses of CHD based on guidelines, an ejection fraction above 45%, and an age range of 18–55 years. The sample consisted of 120 individuals recruited using simple random sampling. To identify patients, we referred to the outpatient clinic records and randomly selected 60 patients from each clinic (Besat and Shahid Rajaei) based on their registration numbers. Then, we planned to fill in the questionnaires at their routine clinic visit for 6 months (from March 2022 to August 2022). On the day of referral to the adult congenital heart disease clinic, we asked patients to complete questionnaires after explaining the study objectives and obtaining their written consent to participate.

The questionnaire comprised two parts. In the first part, demographic questions (age, sex, level of education, employment, and marital status) were asked. In the second part, the QoL was investigated using the Persian version of Ferrans and Powers’ Quality of Life Index (24). The tool, which was developed in 1999, consists of 35 items on different aspects of life, including health and functioning (HF; 15 items), socioeconomics (SE; 8 items), psychological-spiritual (PS; 7 items), and family (FA; 5 items). Individuals indicated their satisfaction level and the importance of that issue on a scale of 1 to 6, from very dissatisfied/unimportant to very satisfied/important. To measure the scores, at first, we subtracted the constant number of 3.5 from all of the satisfaction scores. Hence, the scores changed to −2.5, −1.5, −0.5, +0.5, +1.5, and +2.5. We multiplied these numbers and importance scores together and added these numbers. To avoid the effect of the questions that remained unanswered, we divided this score by the number of questions. The resulting numbers were on a scale from −15 to +15. To remove negative numbers, we added the constant number of +15 to all numbers and got numbers in the range of 0 to 30. We classified them into three categories: undesirable (0–9), relatively desirable (10–19), and desirable (20–30). We used Cronbach’s alpha to evaluate the scale’s reliability, which was approved with a score of 0.86. We classified the severity of CHD according to the guidelines for the management of adults with CHDs developed by the American College of Cardiology (ACC) and the American Heart Association (AHA) (25): greatly complex, moderately complex, and simple. We also evaluated the patient’s heart function (Table 1) by echocardiography. Additionally, the presence of a residual defect, history of arrhythmia, heart surgery and type of surgery (corrective or palliative), functional class (FC), and blood oxygen level were checked from medical records and put into the questionnaire.

**TABLE 1:** Reference value and grading scale for the LVEF.

LV function	LVEF%
Normal	≥55
Mildly reduced	45–55
Moderately reduced	30–44
Severely reduced	<30

LVEF, left ventricular ejection fraction; LV, left ventricle.

### Statistical analysis

We used SPSS software, version 22, for statistical analysis. We applied descriptive analysis to the data and conducted the Chi-squared test to examine the relationship between QoL and variables such as age, duration of the disease, and the number of hospitalizations.

### Ethics statement

This study was approved by the ethical committee of the Rajaei Heart Center (IR.RHC.REC.1400.013). We also ensured that patients had given written informed consent before participating in the study.

## Results

**FIGURE 1:**
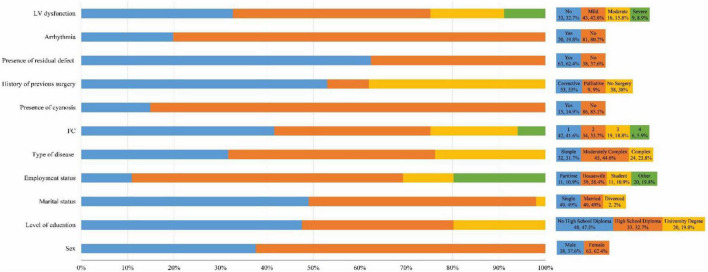
Demographic data of the participants. FC, functional class; LV, left ventricle.

A total of 101 (84% response rate) patients (63 women, 62.4%) with a mean age of 31.05 years participated in the study. The number of single and married participants was equal (49 people, 49%). Moreover, there were two divorced participants (2%). More than half of our patients were house wives (81 patients, 58.9%). A lower level of education was more common in our study, (with 80.2% of them not having a university degree). The most common type of CHD among our study participants was moderately complex (45 patients, 44.6%). Approximately one-third of the study population did not suffer from left ventricle dysfunction, but the most common type of dysfunction was mild (43 patients, 42.6%). The number of people in each FC class decreased from 42 (41.6%) in class one to 6 (5.9%) in class four. A history of previous surgery was a common trait (62 participants, 62%), and most surgeries were corrective (53 participants, 53%). More than 60% of our patients (63) had residual defects. For most individuals, cyanosis (86 patients, 85.1%) and arrhythmia (81 patients, 80.2%) were not observed (Figure 1).

While evaluating the relationship between patient characteristics and QoL, we discovered that only disease-specific factors significantly affected QoL (Table 2).

**TABLE 2:** Relation between patient’s characteristics and quality of life (QoL).

Variable		Quality of life			*P*-value
		**Desir-** **able**	**Relatively desirable**	**Un-** **desirable**	
Sex	Men	25.7	74.3	–	0.22
	Women	16.9	83.1	–	
Marital status	Single	21.7	78.3	–	0.7
	Married	20.5	79.5	–	
	Divorced	–	100	–	
Educational level	No high school diploma	84.1	15.9	–	0.3
	High school diploma	80.6	19.4	–	
	University degree	68.4	31.6	–	
Employment	Part time	30	70	–	0.2
	Housewife	14.3	85.7	–	
	Student	40	60	–	
	Other	22.2	77.8	–	
Type of disease	Simple	20.7	69.2	10.1	0.05
	Moderate	24.4	64.4	11.2	
	Complex	12.5	27.3	60.2	
Type of surgery	Corrective	23.5	55.3	21.2	0.02
	Palliative	12.5	57.5	30.5	
	No surgery	14.7	85.3	–	
Presence of Residual defect	Yes	10.2	89.8	–	0.002
	No	37.1	62.9	–	
FC	1	28.2	71.2	–	0.03
	2	12.5	77.7	10	
	3	16.7	68.2	15.1	
	4	20	50	30	
LV dysfunction	No	35.5	64.5	–	0.06
	Mild	15	85	–	
	Moderate	7.1	92.2	–	
	Severe	11.1	88.9	–	
Presence of cyanosis	Yes	21.4	57.6	21	0.03
	No	20	71	9	
Arrhythmia	Yes	15	85	–	0.5
	No	21.6	78.4	–	

FC, functional class; LV, left ventricle.

### Sex

We had more women in our study than men (63 vs. 38); both sexes had a relatively desirable QoL; however, the ratio of women with better scores of QoL was slightly better than men (83.1 vs. 74.3%). Sex did not have a significant effect on QoL (p-value = 0.224).

### Marital status

The numbers of single and married individuals were the same (49 individuals), and their QoL was very close to each other; we only had two divorced persons in our study, and their scores were both classified as relatively desirable. Marital status was not significantly related to QoL (p-value = 0.726).

### Educational level

Most of our patients had no high school diploma (47.5%); the best QoL was observed in the group with no high school diploma, and the worst was in the group with a university degree. Data for this factor showed that the higher the level of education, the less desirable the life; however, the statistical analysis did not show a statistically significant difference (p-value = 0.372).

### Employment status

Most of our patients were house wives, with the lowest QoL (14.3%), versus students, with the highest number of individuals in the desirable group (40%). The observed difference was not statistically significant (p-value = 0.253).

### Disease characteristics

Simple and moderately complex types of disease had relatively the same QoL, but the complex group had a high ratio of people with an undesirable QoL (60.2%). They also had the lowest ratio of people in the desirable group (12.5%). The effect of the type of CHD was statistically significant (p-value = 0.050).

Most patients had a history of previous surgery, and most surgeries were corrective (51.5%). All patients without a history of previous surgery had a desirable QoL, but 21.2% of patients with previous corrective surgery and 30.5% of patients with previous palliative surgery had undesirable QoL. The type of surgery had a significant effect on QoL (p-value = 0.024).

Another significant factor was the presence of a residual defect (p-value = 0.002). Most of our patients had residual defects (62.4%), and data showed that people with residual defects had significantly lower QoL.

Participants with the lowest FC had the highest ratio of undesirable QoL (30% of the FC group of four); this difference was statistically significant (p-value = 0.031). Dysfunction did not have a significant relationship with QoL (p-value = 0.064). The lowest QoL was observed in the moderate group. This group even had a lower QoL than the group with severe dysfunction (92.2 vs. 88.9%). The presence of cyanosis had a significant impact on QoL (p-value = 0.032), but arrhythmia did not have such an impact (p-value = 0.567).

## Discussion

We performed a descriptive-analytical study on 101 (63 women) adults with CHD to assess the impact of different social, economic, and disease-specific factors on QoL. The statistical analysis did not show any significant relationship between social or economic factors and QoL, but almost all disease-specific factors had a significant relationship with QoL.

Sex, marital status, and educational level constituted the social factors of our study. None of these factors had a significant effect on QoL. The literature is contradictory on the effect of social factors: Jackson et al. (26) had the same results, but Andonian et al. (27) and Rometsch et al. (18) found significantly worse results for female patients. In a study by Apers et al. (13), being married was related to better QoL, but Chen et al. (28) found no relationship between this factor and QoL in their study population. Lower educational level resulted in lower impaired health-related quality of life and psychological adjustment in a study by Rometsch et al. (18); Moons et al. (29) found an association between lower educational level and poorer QoL. The level of education among our participants was low. Cocomello et al. (30) also found a lower level of education among adults with CHD compared to their non-CHD peers.

In addition, it was shown in a previous study that low education can be one of the reasons for these patients not taking medicine, which will ultimately have a negative effect on the disease process and the quality of life of these patients (31).

Students and house wives had the best and worst QoL, respectively, but the statistical analysis did not find a significant difference (*P*-value = 0.212). Most studies (17, 32) showed that employment results in a significantly higher QoL. Eslami et al. (32) indicated that unemployment is the strongest negative factor associated with dissatisfaction.

The results showed that patients with a complex type of disease had worse QoL compared with those with mild and moderate forms of the disease. Some studies (14, 18) showed no significant difference between the QoL of different types of CHD, while others, including a cross-sectional study conducted by Truong et al. (17), reported the severity of the disease as a significant factor; Moons and Luyckx (33), in their review on the QoL of adults with CHD, found that QoL, when evaluated as physical functioning, is worse in complex cases than mild or moderate ones. It can be said that the type of underlying heart disease and its severity is one of the influencing factors on various factors in adult patients. In addition to the relationship between the type of underlying disease and the quality of life of these patients, a study by Westhoff-Bleck et al. showed that there is a relationship between the severity of heart disease and major depression in these patients (10). A history of previous surgery was another significant factor in determining QoL. Patients who had had corrective surgery had the best QoL, possibly because most of these procedures were curative and allowed the patient to potentially have a relatively normal life afterward. The patients without a history of previous surgery had the worst QoL; this could be due to having an inoperable defect or not following the treatment plan. Wang et al. (34) observed a nonsignificant difference between patients with and those without previous surgery, suggesting that both groups had a good capacity to cope with the disease regardless of this factor. Patients without residual defects can experience significantly better QoL (*P*-value = 0.002); these defects can deteriorate a patient’s cardiorespiratory function and can therefore lower the QoL. Furthermore, we should pay attention to the fact that most of the patients with residual defects have complex diseases, and these complex situations will result in a worse outcome (35).

Previous studies (13, 36, 37) showed that higher FC leads to worse QoL. In our study, FC had the same significant impact (*P*-value = 0.032). QoL was significantly lower for cyanotic patients (*P*-value = 0.031); Simko and McGinnis (38), in their study on QoL between different types of CHDs, reported that all the factors related to QoL, except for job, are worse for patients with cyanosis, but none of these factors were significant; they also mentioned that patients with cyanosis are more likely to have no surgical repairs and to be classified as FC I. We can note that these elements as some of the roots of this difference; Bertoletti et al. (39) did not find a significant difference in cyanosis, while Lane et al. (40) reported a significant difference between these two groups (*p* < 0.013).

Patients with no dysfunction and those with mild dysfunction had a better QoL. No arrhythmia had the same effect on QoL, but these (dysfunction, *P*-value = 0.062; arrhythmia, *P*-value = 0.514) were the only two disease-specific factors whose difference was not statistically significant. Disability has been identified (30, 41) as an unfavorable factor for QoL. The study conducted by Loup et al. (42) had the same nonsignificant result; Neiman et al. (43) showed that sexual dysfunction in women is associated with arrhythmias; Irtel et al. (44) studied the impact of arrhythmias on QoL in adults with CHD; atrial tachyarrhythmias and sinus nodal dysfunction, requiring a pacemaker, were the groups with the most impaired QoL; they also reported that patients without arrhythmias had a QoL comparable to that of the general population.

Congenital heart diseases are severe and, in many cases, life-threatening; they need a remarkable amount of additional care, and they also cause mental distress, but most of our patients had a medium or good QoL; multiple studies (19, 45) have shown the same results. We believe that we should search for the roots of this phenomenon in the socio-psychological components; the authors have cited some of the reasons: Bertoletti et al. (19) believed that this was the result of a good family and social support system. They mentioned that these support systems could help create a suitable environment focused on problem-solving and disease adaptation. They also noted that these could lead to better mental health, but it is noteworthy that unpleasant relationships among the members of the family and parental stress can also have adverse effects. Cocomello et al. (28) described disability paradox, sense of coherence, and response shift as three mechanisms that can contribute to this event.

Another result of our study was that almost all of the disease-specific factors were significantly effective on QoL. Given that disease-specific factors can get worse as the disease progresses over time, early diagnosis and treatment are essential for improving QoL; previous studies (46, 47) suggested using pulse oximetry as a simple, inexpensive, and noninvasive screening approach for this purpose. Bruno and Havranek (48) emphasized the importance of a centralized system for reporting positive CHD results, immediate patient evaluation, and suitable follow up for families.

## Limitation

Because this study was conducted in two academic centers, its results cannot be generalized to all patients. In addition to the abovementioned questionnaire, the Ferrans and Powers Quality of Life Index, which is a subjective assessment tool rather than an objective one, may not be able to examine all aspects of the quality of life. There is a possibility of bias in the study due to the presence of confounding factors such as the city of residence.

## Conclusion

To sum up, this study showed that patients with ACHD not only suffer from their main disease but also experience a relatively worse quality of life than the general population. We also found that adults with CHD have a low QoL, which is unrelated to their demographic and socioeconomic factors. This finding suggests that the presence of the disease and its accompanying complications alone can affect these people’s QoL. Therefore, it is crucial to make timely and accurate diagnoses and initiate the appropriate treatment to minimize the complications of the disease as much as possible. This not only reduces the burden of treatment costs but also improves the QoL of these patients.

## Data availability statement

The original contributions presented in this study are included in the article/supplementary material, further inquiries can be directed to the corresponding author.

## Ethics statement

This study was approved by the Ethical Committee of Rajaei Heart Center (IR.RHC.REC.1400.013). The patients/participants provided their written informed consent to participate in this study.

## Author contributions

MA, ZK, ZA, and AS drafted the manuscript. MA and ZK finalized it. All authors read and approved the final version of the manuscript.
